# Is the CTS5 a helpful decision-making tool in the extended adjuvant therapy setting?

**DOI:** 10.1007/s10549-023-07186-6

**Published:** 2024-01-25

**Authors:** Kerstin Wimmer, Dominik Hlauschek, Marija Balic, Georg Pfeiler, Richard Greil, Christian F. Singer, Stefan Halper, Günther Steger, Christoph Suppan, Simon P. Gampenrieder, Ruth Helfgott, Daniel Egle, Martin Filipits, Raimund Jakesz, Lidija Sölkner, Christian Fesl, Michael Gnant, Florian Fitzal

**Affiliations:** 1 Department of General Surgery, Division of Visceral Surgery, Medical University of Vienna, Vienna, Austria; 2 Austrian Breast & Colorectal Cancer Study Group, Vienna, Austria; 3 Department of Oncology, Medical University of Graz, Graz, Austria; 4 Comprehensive Cancer Center, Medical University of Vienna, Vienna, Austria; 5 Department of Gynecology and Obstetrics, Medical University of Vienna, Vienna, Austria; 6 Department of Internal Medicine I, Medical University of Vienna, Vienna, Austria; 7 Department of Internal Medicine III with Haematology, Medical Oncology, Haemostaseology, Infectiology and Rheumatology, Oncologic Center, Paracelsus Medical University Salzburg, Salzburg, Austria; 8 Salzburg Cancer Research Institute-CCCIT, Salzburg, Austria; 9 Cancer Cluster Salzburg, Salzburg, Austria; 10 Department of Surgery, Ordensklinikum Linz - Sisters of Charity, Linz, Austria; 11 Department of Gynaecology, Medical University Innsbruck, Innsbruck, Austria; 12 Center for Cancer Research, Medical University of Vienna, Vienna, Austria; 13 Department of Surgery, Regional Hospital Wiener Neustadt, Wiener Neustadt, Austria

**Keywords:** Breast cancer, CTS5 score, Adjuvant, Extended endocrine therapy, Prediction

## Abstract

**Purpose:**

The Clinical Treatment Score post-5 years (CTS5) is an easy-to-use tool estimating the late distant recurrence (LDR) risk in patients with hormone receptor-positive breast cancer after 5 years of endocrine therapy (ET). Apart from evaluating the prognostic value and calibration accuracy of CTS5, the aim of this study is to clarify if this score is able to identify patients at higher risk for LDR who will benefit from extended ET.

**Methods:**

Prognostic power, calibration, and predictive value of the CTS5 was tested in patients of the prospective ABCSG-06 and -06a trials (*n* = 1254 and 860 patients, respectively). Time to LDR was analyzed with Cox regression models.

**Results:**

Higher rates of LDR in the years five to ten were observed in high- and intermediate-risk patients compared to low-risk patients (HR 4.02, 95%CI 2.26–7.15, *p* < 0.001 and HR 1.93, 95%CI 1.05–3.56, *p* = 0.035). An increasing continuous CTS5 was associated with increasing LDR risk (HR 2.23, 95% CI 1.74–2.85, *p* < 0.001). Miscalibration of CTS5 in high-risk patients could be observed. Although not reaching significance, high-risk patients benefitted the most from prolonged ET with an absolute reduction of the estimated 5-year LDR of − 6.1% (95%CI − 14.4 to 2.3).

**Conclusion:**

The CTS5 is a reliable prognostic tool that is well calibrated in the lower and intermediate risk groups with a substantial difference of expected versus observed LDR rates in high-risk patients. While a numerical trend in favoring prolonged ET for patients with a higher CTS5 was found, a significantly predictive value for the score could not be confirmed.

**Clinical trial registration:**

ABCSG-06 trial (NCT00309491), ABCSG-06A7 1033AU/0001 (NCT00300508).

## Introduction

Recent research gives evidence that in hormone receptor (HR)-positive breast cancer (BC), the risk of recurrence remains elevated up to three decades after primary diagnosis. Factors that increase the hazard for late BC recurrence are larger tumor size (> 2 cm), lymph node positivity and estrogen receptor-positivity [1, 2]. These insights might justify extended surveillance aiming to detect early local recurrences [3] as well as prolonged or more efficient novel treatments in these patients. Extending adjuvant endocrine therapy (ET) beyond 5 years has shown beneficial effect on oncological outcome [4–8]. According to the St. Gallen consensus guidelines 2021 patients with high-risk BC such as lymph node-positive disease at diagnosis and patients with higher risk genomic signature scores should be advised to extended ET [4, 9–11].

Beside expensive multigenomic tests such as Oncotype DX [12], Endopredict [13], Prosigna [14], MammaPrint [15] or Breast Cancer Index [16] that predict distant recurrence (DR) at the time of diagnosis (or operation), a cheaper Clinical Treatment Score post-5 years (CTS5) has been developed to identify HR-positive BC patients at higher risk for late distant recurrence (LDR) after 5 years of ET. Its calculation is cost-free and—by using only parameters that are measured in all patients at the time of diagnosis or operation-, simple.

Since the rationale for extended ET is a persisting recurrence risk in patients with HR-positive BC, any potential predictive value of CTS5 might be helpful to guide this decision-making process [17]. In its original training cohort—the Arimidex, Tamoxifen, Alone or in Combination (ATAC) trial—as well as in its validation cohort—the breast international group (BIG 1–98) trial - the CTS5 seemed to be a promising tool to identify patients at low risk of recurrence.

In this study, we validate the prognostic performance and calibration accuracy of the CTS5 in patients of the ABCSG-06 trial, who did not receive extended aromatase inhibitor (AI)-treatment. Further, we evaluate the predictive value of the CTS5 in patients of the subsequent ABCSG-06a trial, in which patients were either randomized to no prolonged therapy or three additional years of anastrozole.

## Methods

In the prospective ABCSG-06 trial (NCT00309491), 2020 patients were randomly assigned to either five years of tamoxifen or to tamoxifen in combination with aminoglutethimide for the first two years of ET. After a median follow-up of 5.3 years, no significant differences in disease-free or overall survival were found between patients with or without 2 years of aminoglutethimide in addition to tamoxifen for 5 years [18].

In ABCSG-06a (NCT00300508), 860 patients, who were recurrence-free after 5 years, were prospectively randomized to additional 3 years of anastrozole or no further treatment. After a median follow-up of 5.2 years, significantly lower rates of recurrence (locoregional, contralateral or distant metastasis) were observed in women who underwent extended ET therapy [5].

According to the pivotal paper published in 2018 by Dowsett et al., the calculation of the CTS5 score as well as the separation into three groups was performed by using the pre-defined cut-off values [17]. The calculation was performed by using the formula “CTS5 = 0.438 × nodes + 0.988 × (0.093 × size + 0.001 × size^2^ + 0.375 × grade + 0.017 × age)”, where ‘nodes’ represent five ordinal categories (0 for N0, 1 for one positive, 2 for two to three positive, 3 for four to nine positive, and 4 for more than nine positive nodes); ‘size’ represents the continuous tumor size in millimeters and ‘size^2^’ represents the quadratic tumor size (tumor size is capped at 30 mm); ‘grade’ represents 3 ordinal categories (1 for G1, 2 for G2 and 3 for G3) and ‘age’ represents numerical age at randomization. Risk cut-offs are CTS5 < 3.13 for low risk, CTS5 3.13 to 3.86 for intermediate risk and CTS5 > 3.86 for high risk [17]. The three risk groups were defined as risk of DR in the years 5 to 10 in: low < 5%, intermediate 5% to 10%, and high risk > 10%.

The primary objective was to evaluate the prognostic performance of the continuous CTS5 in all patients of the ABCSG-06 cohort without extended therapy (i.e. ABCSG-06a extended AI arm). Patients with missing tumor data, DR events or discontinuation of the study within the first 5 years were excluded. In 89 patients (7.1%) data about tumor grade was missing (“GX”). As G2 was by far the most frequent class (57%) and as there are no strong predictors for grading, a logistic regression imputation model using other clinical factors as well as age predicted G3 only once for a GX case whereas all other GX cases were predicted to be G2. Therefore, all patients with “GX” were assigned to G2. The observation period started five years after randomization and ended at maximum follow-up. Secondary objectives included the evaluation of the categorical CTS5, the calibration accuracy as well as the predictive power of the CTS5. Calibration was assessed in ABCSG-06 patients without extended therapy by comparing predicted with observed LDR rates. Evaluation of the predictiveness was performed in all ABCSG-06a patients and observation period started from randomization into this substudy.

The endpoint was time to LDR, defined as metastatic disease excluding contralateral, locoregional as well as ipsilateral recurrence, after five recurrence-free years postoperatively. The 5- to 10-year DR risk was calculated for ABCSG-06 patients who were disease-free 5 years beyond randomization. For patients who were further randomized to the ABCSG-06a trial, the LDR was assessed in the years 0 to 5, equivalent to the 5- to 10-year interval in ABCSG-06.

Patients with no LDR at last follow-up or death were censored. All risk estimates are hypothetical in nature, because deaths are ignored (i.e. the “true” DR risk—accounting for death—would be smaller). In addition, a sensitivity analysis was performed to check the possible influence of a documentation bias (i.e., it was hypothesized that documentation of DR events after a preceding non-DR event was less common). However, censoring for earlier non-DR events did not change the results (e.g., the 10-year risk was identical).

### Statistical analysis

Prognostic analyses were carried out with proportional hazard Cox models. Hazard Ratios (HR) and their corresponding 95% confidence intervals (CI) are reported. The proportional hazards assumption and the functional form of the continuous CTS5 were assessed. No violations have been found. Concordance indices (c-index) according to Harrell et al. are also reported [19]. Furthermore, Kaplan Meier (KM) curves including pointwise 95% CIs were evaluated for CTS5 risk categories.

The calibration of CTS5 was examined with a calibration plot. Due to sample size limitations all patients were grouped into 5 quantiles based on their predicted risk. The KM estimates of each quantile were compared with the median predicted risk.

The predictive power of CTS5 was assessed on the relative (HR scale) and on the absolute scale (difference in LDR risk estimates). Cox models with interaction terms of treatment arm and CTS5 as well as KM estimates were derived. A *p*-value of < 0.05 was considered statistically significant. Analyses were carried out using SAS software (version 9·4).

## Results

**Fig. 1:**
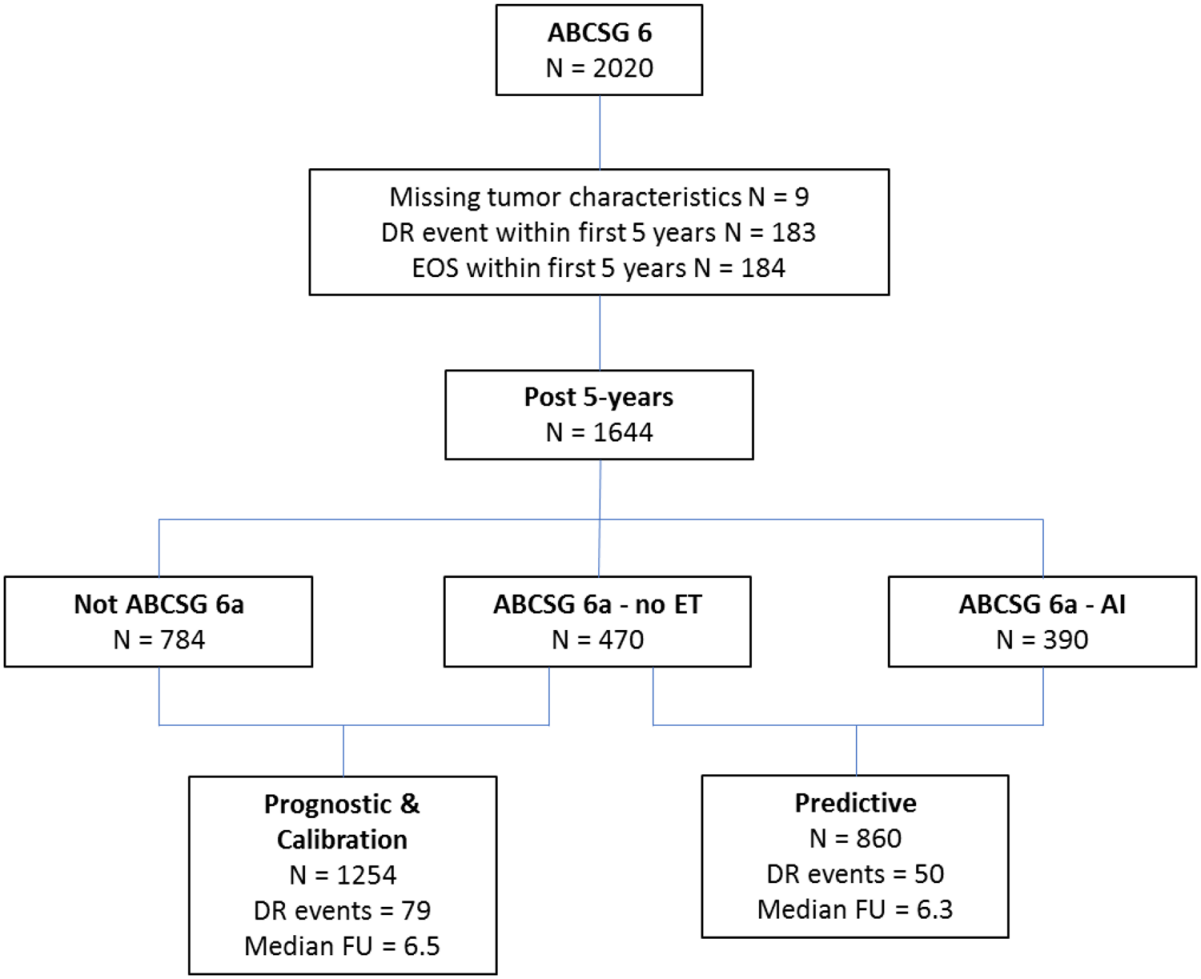
Flow chart showing patient selection of the ABCSG-6 trial. For the investigation of the prognostic validation and the calibration of the CTS5 1254 patients were selected, who didn’t receive extended ET. Side note: Median FU between “Not ABCSG-6a” and “ABCSG-6a” was very similar (6.3 vs 6.6years). *DR* Distant recurrence, *FU* Follow up, *EOS* End of study, Graphic was created with power point (MS office 365)

The ABCSG-06 trial included 2020 patients of which nine had missing tumor characteristics, 183 had a LDR event within the first 5 years and 184 reached the end of study within the first 5 years, resulting in 1644 patients in whom a valid calculation of the CTS5 score was performed (Fig. 1). Of these patients, 860 were randomized within the ABCSG-06a trial to either no further therapy (*n* = 470) or to prolonged ET with 3 years additional anastrozole (*n* = 390).

For the evaluation of the prognostic performance, 1254 patients without prolonged ET were included. After a median follow-up of 6.5 years, starting after 5 years of ET (11.5 years after randomization), 79 patients had a DR event, translating to 5- to 10-year DR-free rate of 95.0%. Table 1. shows baseline characteristics of the primary analysis set by CTS5 risk categories.

**Table 1:** Patient’s characteristics according to CTS5 risk categories in 1254 patients without extended ET

	Low risk *N* = 507	Intermediate risk *N* = 440	High risk *N* = 307	Total *N* = 1254
**Age (years)**				
*N*	507	440	307	1254
Mean (SD)	62.5 (7.7)	64.6 (7.4)	64.3 (7.7)	63.7 (7.6)
Median (Q1–Q3)	63.0 (56.0–69.0)	65.0 (59.0–70.0)	65.0 (58.0–70.0)	65.0 (58.0–70.0)
Min–Max	41.0–79.0	47.0–80.0	43.0–79.0	41.0–80.0
**Tumor size (cm)**				
*N*	507	440	307	1254
Mean (SD)	1.2 (0.4)	2.1 (0.9)	2.8 (1.2)	1.9 (1.1)
Median (Q1–Q3)	1.2 (0.9–1.5)	2.0 (1.6–2.5)	2.5 (2.0–3.2)	1.7 (1.2–2.4)
Min–Max	0.2–2.5	0.7–8.0	0.8–10.0	0.2–10.0
**T-stage**				
pT1a	20 (3.9%)	0	0	20 (1.6%)
pT1b	168 (33.1%)	21 (4.8%)	5 (1.6%)	194 (15.5%)
pT1c	297 (58.6%)	204 (46.4%)	72 (23.5%)	573 (45.7%)
pT2	22 (4.3%)	209 (47.5%)	210 (68.4%)	441 (35.2%)
pT3	0	6 (1.4%)	20 (6.5%)	26 (2.1%)
**Positive nodes** (*n*)				
*N*	507	440	307	1254
Mean (SD)	0.1 (0.3)	0.4 (0.8)	3.6 (3.9)	1.1 (2.5)
Median (Q1–Q3)	0.0 (0.0–0.0)	0.0 (0.0–1.0)	2.0 (1.0–5.0)	0.0 (0.0–1.0)
Min–Max	0.0–3.0	0.0–4.0	0.0 to 21.0	0.0–21.0
**N-stage**				
pN0	469 (92.5%)	321 (73.0%)	42 (13.7%)	832 (66.3%)
pN1	38 (7.5%)	116 (26.4%)	163 (53.1%)	317 (25.3%)
pN2	0	3 (0.7%)	77 (25.1%)	80 (6.4%)
pN3	0	0	25 (8.1%)	25 (2.0%)
**Grade**				
G1	157 (31.0%)	32 (7.3%)	9 (2.9%)	198 (15.8%)
G2	280 (55.2%)	285 (64.8%)	148 (48.2%)	713 (56.9%)
G3	34 (6.7%)	87 (19.8%)	133 (43.3%)	254 (20.3%)
GX	36 (7.1%)	36 (8.2%)	17 (5.5%)	89 (7.1%)

Patients had a mean age of 63.7, the majority had pT1 (62.8%) and pN0 (66.3%) tumors. Low-risk patients had smaller tumors with earlier T- and N-stages and lower tumor grade than patients in the higher risk groups.

### Prognostic performance of the continuous CTS5

The prognostic performance of the continuous CTS5 showed that an increasing score was associated with an increased LDR risk (HR 2.23, 95%CI 1.74–2.85, *p* < 0.001). The c-index of 0.68 indicated a moderate prognostic power. In a sensitivity analysis censoring at year 10 from randomization to the ABCSG-06 study the continuous CTS5 remained prognostic (HR 2.10, 95%CI 1.57–2.81, *p* < 0.001).

In a subgroup analysis, the prognostic value of the continuous CTS5 was investigated according to the nodal status. No differential effect could be shown (interaction *p*-value = 0.467), however, both HR effect sizes were reduced (node-negative: HR 2.12, 95%CI 1.01–4.43; node-positive: HR 1.58, 95%CI 1.08–2.30).

### Prognostic performance of the categorical CTS5

In the ABCSG-06 cohort 307 patients (24.5%) were assigned to the high-risk group, 440 patients (35.1%) to the intermediate-risk group and 507 patients (40.4%) to the low-risk group. Patients in the high-risk group had a much higher rate of LDR than low-risk patients (HR 4.02, 95%CI 2.26–7.15, *p* < 0.001) as well as intermediate-risk patients compared to low-risk patients (HR 1.93, 95%CI 1.05–3.56, *p* = 0.035).

**Fig. 2:**
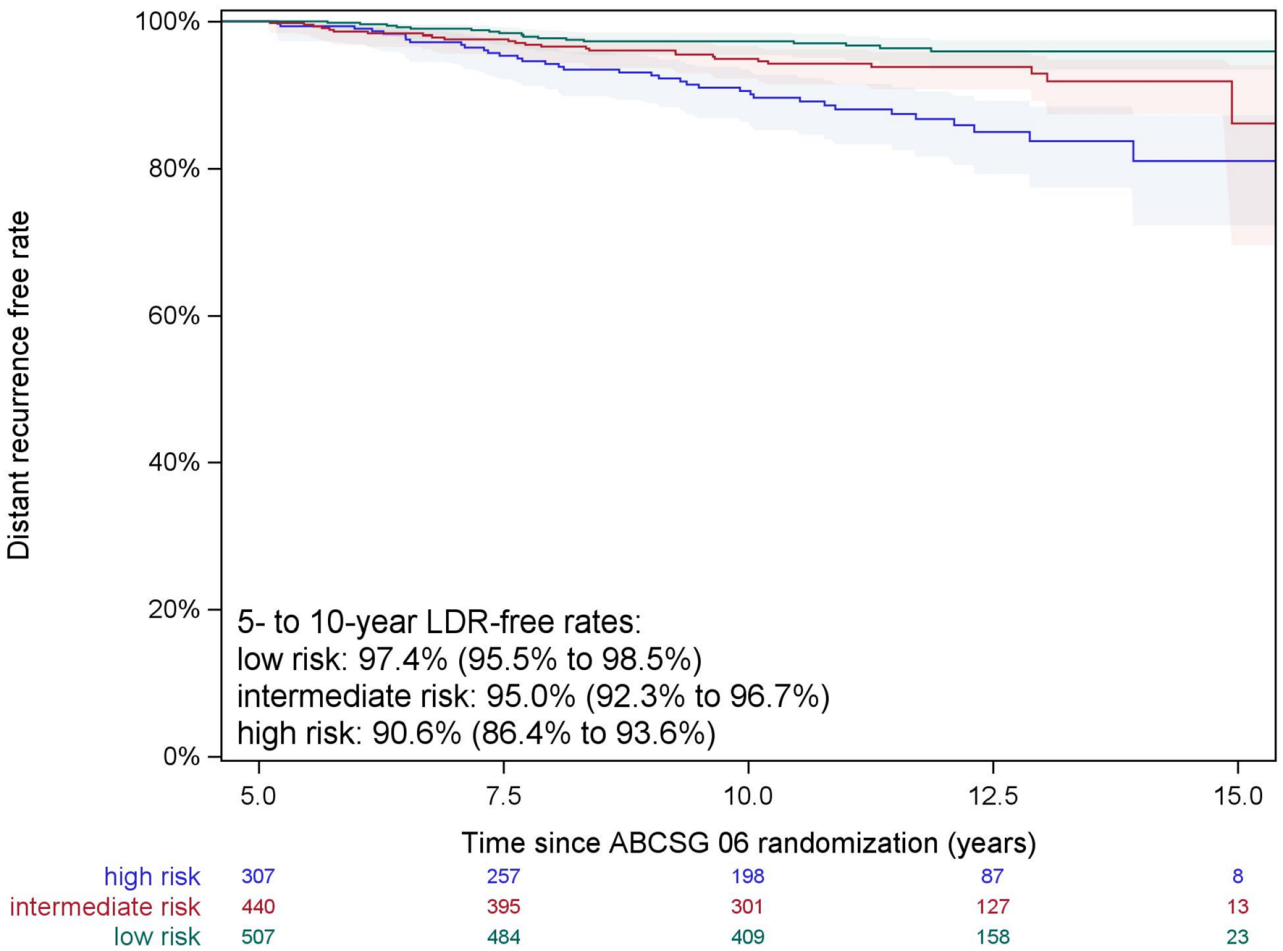
5- to 10-year DR-free survival curves with 95% confidence interval bands according to CTS5 risk categories

A 5- to 10-year LDR-free survival of 90.6% (95%CI 86.4–93.6%) in the high-risk, 95.0% (95%CI 92.3–96.7%) in the intermediate-risk and 97.4% (95%CI 95.5–98.5%) in the low-risk group was observed. The KM curves for the three CTS5 risk groups are shown in Fig. 2.

In node-negative patients, the HR for a LDR event in high- versus low-risk patients was 1.84 (95%CI 0.42–8.09), whereas the HR for intermediate- versus low-risk patients was 1.45 (95%CI 0.68–3.08). In node-positive patients a similar trend was observed. The HRs of high- versus low-, and intermediate versus low-risk patients were similar with 1.82 (95%CI 0.56–5.93) and 1.57 (95%CI 0.45–5.50), respectively. The interaction test showed no differential effect in those subgroups (*p* = 0.990). Data is shown in Appendix Fig. 5 and Table 3

### Calibration of CTS5

**Fig. 3:**
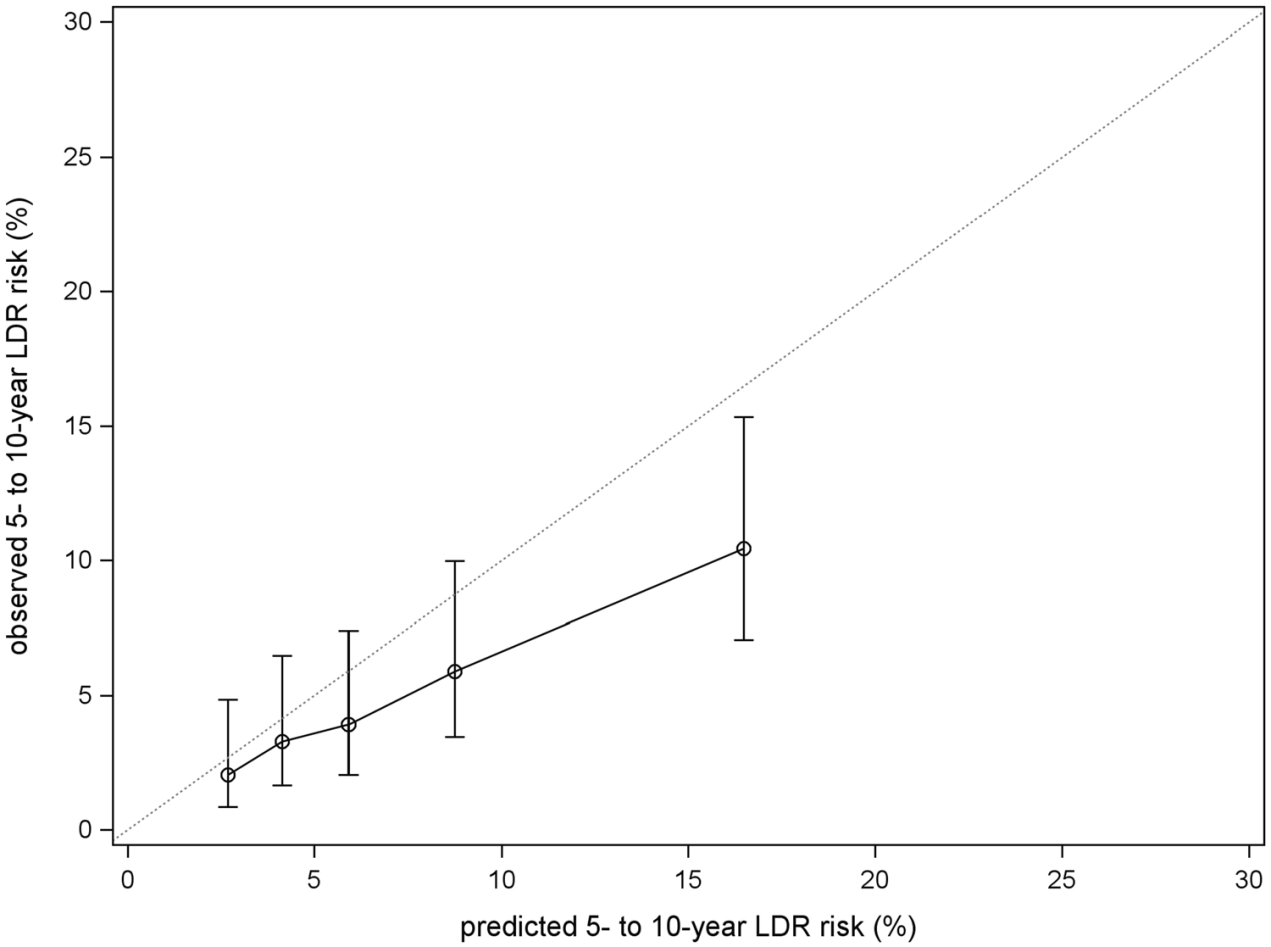
Calibration plot of CTS5 score comparing the observed LDR risk (estimated with Kaplan Meier methodology) with predicted risks. Patients were combined into 5 groups according to their predicted LDR risk. 95% confidence intervals are shown

Comparing the predicted versus the observed LDR risk showed that the higher the predicted risk, the less accurate the score was (see Fig. 3). In the lowest CTS5-quantile (*n* = 251), the DR risk-difference was − 0.6% (median predicted risk = 2.7%, KM risk = 2.0% (0.9%–4.8%)) whereas a risk difference of − 6.0% was observed in the highest CTS5-quantile (*n* = 252, median predicted risk = 16.5%, KM risk = 10.4% (7.1%–15.3%)).

### Predictive performance of CTS5

**Fig. 4:**
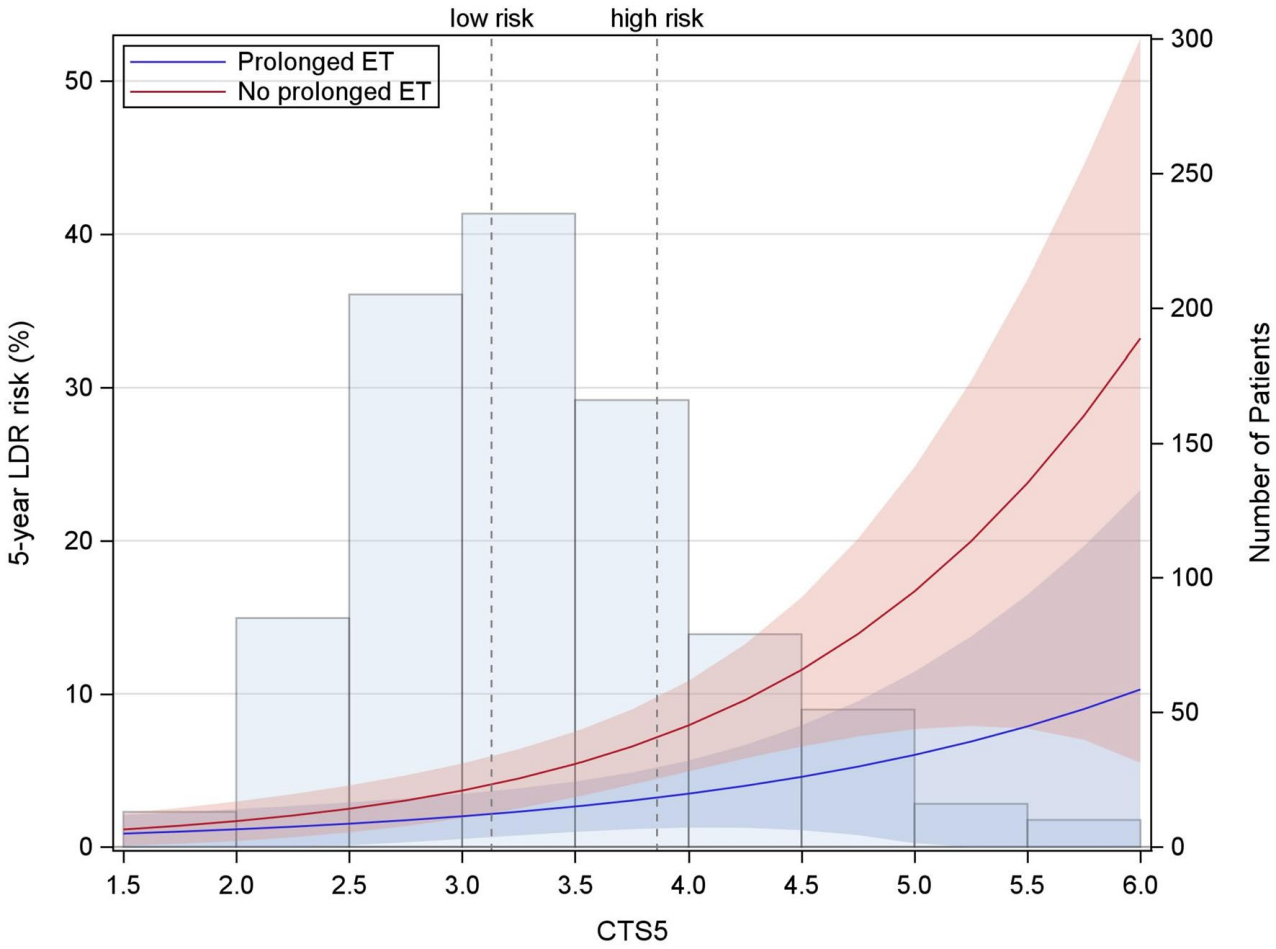
Five-year LDR risk (starting at randomization to ABCSG-06a) according to continuous CTS5 score in patients with additional 3 years of anastrozole (prolonged; blue line) and not prolonged (red line) therapy. The confidence bands reflect the 95% pointwise confidence intervals. The semi-transparent bars reflect the number of patients in each CTS5 score group

The predictive performance for both, the continuous as well as the categorical CTS5, was investigated in 860 patients (LDR events = 50) with a median follow-up of 6.3 years starting from randomization to ABCSG-06a. The predictive performance of the continuous CTS5 could not be statistically confirmed on the relative scale (interaction *p* = 0.497), although a numerical trend in favoring prolonged ET for patients with a higher CTS5 was found (see Table 2a). A difference in the 5- to 10-year LDR risk was observed between patients with and without extended treatment (see Fig. 4).

**Table 2:** Predictive performance of the continuous (a) and the categorical (b) CTS5 score

a				
	Relative scale	Absolute scale (5-year DR risk^b^)		
CTS5	HR^1^ (95% CI)	No prolonged ET	Prolonged ET^c^	Difference
	Interaction p-value *p* = 0.497			
2	0.84 (0.22, 3.15)	1.7% (0.4%, 3.0%)	1.1% (0.0%, 2.5%)	− 0.5 (− 2.4, 1.3)
3	0.66 (0.30, 1.45)	3.7% (1.9%, 5.4%)	2.0% (0.5%, 3.5%)	− 1.7 (− 4.0, 0.6)
4	0.53 (0.28, 0.98)	7.9% (4.9%, 10.8%)	3.5% (1.3%, 5.6%)	− 4.5 (− 8.1, − 0.8)
5	0.42 (0.15, 1.17)	16.7% (7.7%, 24.8%)	6.0% (0.2%, 11.4%)	− 10.7 (− 20.9, − 0.5)
6	0.33 (0.06, 1.68)	33.2% (5.5%, 52.8%)	10.3% (0.0%, 23.3%)	− 22.9 (− 50.0, 4.2)

b				
	Relative scale	Absolute scale (5-year DR risk^d^)		
CTS5 risk group	HR^a^ (95% CI)	No prolonged ET	Prolonged ET^c^	Difference
	Interaction p-value *p* = 0.644			
low risk	0.89 (0.20, 3.99)	1.5% (0.5%, 4.7%)	0.6% (0.1%, 4.4%)	− 0.9 (− 3.0, 1.2)
intermediate risk	0.40 (0.15, 1.09)	7.1% (4.1%, 12.2%)	3.1% (1.2%, 8.0%)	− 4.0 (− 8.9, 0.9)
high risk	0.62 (0.26, 1.49)	12.0% (6.8%, 20.6%)	5.9% (2.5%, 13.6%)	− 6.1 (− 14.4, 2.3)

The predictive value could not be confirmed although a trend of beneficial outcome of patients with extended ET and a higher CTS5 score was observed. For example, patients with a CTS5 score of 6 and prolonged ET had a three-time smaller risk of DR than patients with the same CTS5 without prolonged therapy. The interaction p-value (relative scale) was neither significant regarding the continuous CTS5 nor regarding the categorical CTS5.

^a^Hazard Ratio for 3-years anastrozole versus no prolonged ET

^b^Estimated with univariate Cox regression models separately by treatment arm

^c^Three years of additional anastrozole

^d^Estimated with Kaplan Meier methodology

Similar to the trend on the relative scale, the higher the score, the greater the absolute difference of LDR risks between patients with and without extended ET: In patients with a CTS5 score of 2, the LDR rates only differed minimally between extended and non-extended therapy (1.1% versus 1.7%), whereas for a CTS5 score of 6, patients seemed to derive a somewhat greater benefit of extended ET [absolute difference of − 22.9% (95%CI − 50.0 to 4.2)].

With respect to the predictive performance of the categorical CTS5, no significant predictive power was found on the relative scale (interaction *p* = 0.644, see Table 2b). On the absolute scale, numerically decreased risk rates for LDR in years 5 to 10 were found in patients who underwent extended anastrozole for three years after initial 5 years of ET across all three risk categories. However, no risk category benefitted significantly from prolonged ET, with the highest numerically risk reduction at 5 years in high-risk patients (− 6.1%, 95%CI − 14.4 to 2.3). Taken together, although the predictive value could not be confirmed, a trend of beneficial outcome of patients with higher CTS5 scores—either continuous or categorical—, who underwent extended ET, was clearly observable. 

## Discussion

A variety of prognostic multigene signatures is available to estimate the risk of recurrence in women with HR-positive BC. All of them are expensive, not easily available, and it takes a certain processing time until the results can be obtained. As ER-positive BC is known to recur even many years after the end of adjuvant ET, there is still a great demand for a tool accurately predicting the risk of (late) recurrence.

CTS5 is a simple score, which is available as an online tool, but can also be calculated with its formula. It is supposed to estimate the risk of recurrence in year five to ten after completing five years of ET recurrence-free in HR-positive BC patients. Lately, an external validation in the IDEAL (*n* = 1591) and TEAM (*n* = 5895) trial cohorts was performed [20]. While the prognostic power of the score was confirmed in these validation studies, its predictive value was deemed questionable.

When discussing the prognostic and predictive value of the CTS5 it is important to be aware of the different capability of “prognostic” and “predictive” tools. Prognostic markers are clinical or biological factors that relate to the inherent characteristics of a disease, objectively predicting the outcome of a patients and estimating the risk—irrespective of the administered treatment. In contrary, a predictive marker is able to predict the effect of a certain treatment, compared to their condition at baseline. Mostly, biomarkers have both abilities to some extent, but one commonly dominates [21, 22]. Sechidis et al. stated that mistakenly assuming a biomarker to be predictive, when it is in fact largely prognostic may result in overestimating the benefits of the treatment for a subset of the population [21]. With this in mind, it shouldn’t be considered automatically that patients with worse prognosis will benefit the most of a distinct treatment. However, it is also clear that patients with a worse prognosis have also more potential for improvement meaning that given a constant relative treatment benefit in groups of patients with different risk of disease also means a greater absolute treatment benefit for the higher risk patients.

Here, we present another external validation of the CTS5 in the large ABCSG-06 and -06a study cohorts in regard to its prognostic and predictive performance as well as its calibration accuracy. We confirm the prognostic value of the CTS5 regarding LDR in our ABCSG06/6a study cohort including 1254 patients. Both, the continuous as well as the categorical CTS5 precisely discriminate between low-, intermediate- and high-risk patients.

In a comparable but smaller study, Villasco et al. confirmed the prognostic value of the CTS5 in a retrospective cohort with 603 patients: They found that high-risk patients had a 4-fold higher rate of late DR than patients in the low-risk group [23]. With a HR of 4.02 for a LDR event in high-risk patients we could confirm these findings.

Knowing that the N-stage is a component of the CTS5-formula, a subgroup analysis in our study cohort showed that the prognostic performance of either the continuous or the categorical CTS5 is reduced as soon as patients were stratified according to nodal stage. This indicates that the prognostic information of the CTS5 is mainly driven by nodal status. Dowsett et al. described that almost all patients of the low-risk group were node-negative whereas almost all of the high-risk group were node-positive. Only two of 133 low-risk patients with one to three positive lymph nodes had a DR event between years 5 and 10 in this trial [17]. This just emphasizes once more the paramount prognostic importance of nodal status. Beside nodal status, tumor size, age and tumor grade flow into CTS5 calculation. The predictive strength of those partially non-biological parameters might be debatable, since big randomized control trials as MA.17 [24], MA.17R [7] and NSABP-33 [25] couldn’t determine a subgroup benefiting more than others from extended ET. Only the IDEAL [26] and DATA trial [27] found beneficial effects of extended ET in patients with high-risk tumor characteristics such as nodal positivity (IDEAL and DATA) or ≥ pT2 tumors (DATA).

By using the pre-defined risk cut-offs, the score categorizes in patients with low (5% LDR in year 5 to 10 after diagnosis), intermediate (5–10% recurrences) and high risk of recurrence (> 10% recurrences). In the original CTS5 training cohort—the ATAC trial—as well as in the validation cohort—the BIG 1–98 trial—high-risk patients faced a risk of LDR of 17.3 to 20.3% at year 10 [17]. In comparison, patients, who were assigned to high-risk group by using the CTS5 in our cohort, had a 10-year LDR risk of 9.4%, which would imply intermediate risk of recurrence. This is noteworthy since patients of our cohort anyway were mostly assigned to the low or intermediate risk group. In our cohort, patients had a higher median age (65 years) when compared to the ATAC (64 years) or BIG 1–98 (61 years) cohorts [17]. Since age is also integrated in the CTS5-formula, it might explain that a higher proportion of our patients was classified as high-risk due to their age rather than to their biological factors. Additionally, a higher proportion of G3 were found in the high-risk groups of the ATAC (48.4%) and the BIG 1–98 (45.8%) cohort [17] than in the here presented study (43.3%). However, it is very unlikely that the statistical method of imputation of the GX cases (7.1%) would explain the observed difference in LDR in high-risk patients. Consequently, reconsidering the pre-defined cut-offs for the respective risk category—e.g. increasing the cut-off value for the high-risk group—might be of particular interest.

The calibration accuracy of the CTS5 was already discussed controversially in the literature. We observed that in low-risk patients, the CTS5 is a well-calibrated tool estimating LDR risk practically equivalent to the observed rates. Increasing inaccuracy of the score was found the higher the predicted risk was. Similarly, Noorhoek et al. found an overestimation of late DR in high-risk patients in the TEAM and IDEAL trials: they described a difference of 10% between predicted (29%) and observed (19%) risk in high-risk patients [20]. Dowsett criticized that predominantly patients with extended ET were included [28]. In our study, the CTS5 calibration was assessed in patients without extended ET. A recent validation study in an unselected cohort confirmed that the high-risk group had significantly higher expected than observed DR rates. Excluding those with ET over 60 months resulted in a discordance of this effect [29]. Thus, clinical decision-making on the basis of the CTS5 score, classifying a patient as high-risk for recurrence, might be done cautiously. In these cases, additionally performed multigenomic tests might be recommended to reassure the high-risk profile.

Nevertheless, the problem of risk over- and underestimation is also not fully resolved when multigenomic tests are applied. In sometimes highly heterogeneous tumors, gene expression panels (GEP) of a single core might lead to a wrong assessment. In up to 25% other risk categories were found when different sections of the tumor were used [30]. In general, GEPs as Endopredict are highly prognostic [31], and even add prognostic information to the CTS5 score [32]. Information regarding overestimation of risk in the high-risk group is scarce but there is evidence that even different GEPs classify high-risk patients discordantly [33].

Regarding the predictive performance of the continuous CTS5 we found a numerical trend without statistical significance: patients with extended ET and a (maximum) CTS5 score of 6, had an almost 23 percentage points lower 5-years LDR risk than patients without extended ET with a similar CTS5 score. The circumstance of not reaching significance might be caused by the low numbers of LDR events as well as the low number of high-risk patients in the predictive cohort.

Villasco et al. reported that the CTS5 predicted ET extension benefits in pre- and postmenopausal patients in a retrospective analysis including 783 patients [34]. In comparison to our study, where 45% of patients underwent additional 3 years of anastrozole, in Villasco’s trial only 23% (*n* = 180) received extended ET. They observed that high-risk patients with extended ET had reduced risk for late DR by 63%. Since no formal interaction test was reported, the results must be interpreted carefully. Further, the decision of therapy extension was based on clinicians’ choice and was not part of a prospective randomization. Lee et al. expressed their concerns about the predictive performance of the score in premenopausal women. In their trial, the CTS5 underestimated risk in premenopausal patients [35].

Limitation of the here presented study is the small event size, which impedes the detection of small to moderate—but clinically meaningful—predictive interaction effects.

Taken together, the CTS5 it is a well calibrated prognostic tool in low- and intermediate risk groups. In high-risk patients, a greater difference between predicted and observed DR rates has to be expected. Nonetheless, we could not confirm that the CTS5 reliably predicts benefits from extended therapy although a beneficial effect of extended ET was observable in high-risk patients. When using the continuous CTS5 at least a numerical trend was observable, but more events are needed to derive a definitive conclusion. Thus, the continuous CTS5 should preferably be chosen.

## Figures and Tables

**Fig. 5:**
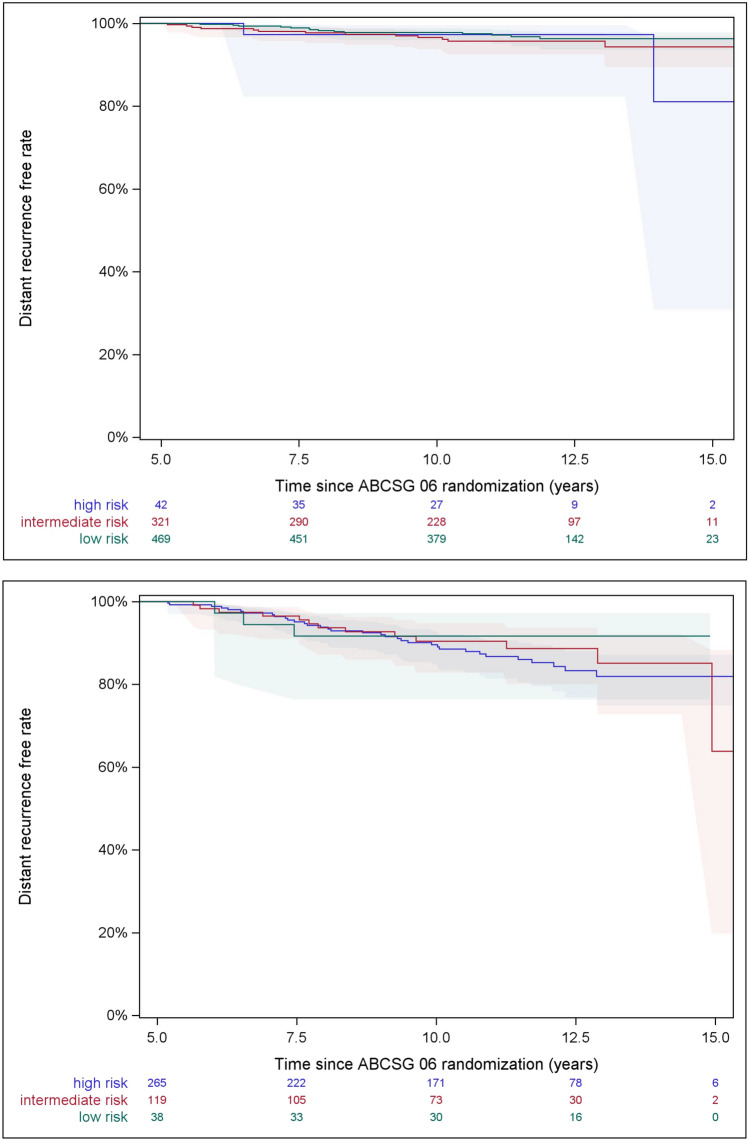
DR-free survival curves according to CTS5 risk categories stratified by nodal status. **A1** Nodal negative, **A2** nodal positive

## Data Availability

The dataset used is not publicly available as it may contain information that would compromise patient consent. Please contact the corresponding author for more information. The published results of the two datasets of the randomized, prospective ABCSG-06 (NCT00309491) and ABCSG-06A/ 1033AU/0001 (NCT00300508) trial conducted by the Austrian Breast Cancer and Colorectal Study Group (ABCSG) are online available (ABCSG-06: PMID: 12637461; ABCSG-06A: PMID: 18073378).

## Appendix

**Table 3:** Univariable Cox regression models separately for nodal status negative and positive patients

Subgroup	*N*	Events	Categories	Cox model	
				Hazard ratio (95% CI)	*p*-value
Nodal status	1254	79			
Negative	832	29	High risk vs low risk	1.84 (0.42–8.09)	0.421
			Intermediate risk vs low risk	1.45 (0.68–3.08)	0.335
Positive	422	50	High risk vs low risk	1.82 (0.56–5.93)	0.322
			Intermediate risk vs low risk	1.57 (0.45–5.50)	0.485

Interaction *p*-value = 0.990

See Table 3 and Fig. 5
