# Correction: Is the CTS5 a helpful decision-making tool in the extended adjuvant therapy setting?

**DOI:** 10.1007/s10549-024-07351-5

**Published:** 2024-05-05

**Authors:** Kerstin Wimmer, Dominik Hlauschek, Marija Balic, Georg Pfeiler, Richard Greil, Christian F. Singer, Stefan Halper, Günther Steger, Christoph Suppan, Simon P. Gampenrieder, Ruth Helfgott, Daniel Egle, Martin Filipits, Raimund Jakesz, Lidija Sölkner, Christian Fesl, Michael Gnant, Florian Fitzal

**Affiliations:** 1 Department of General Surgery, Division of Visceral Surgery, Medical University of Vienna, Vienna, Austria; 2 Austrian Breast & Colorectal Cancer Study Group, Vienna, Austria; 3 Department of Oncology, Medical University of Graz, Graz, Austria; 4 Comprehensive Cancer Center, Medical University of Vienna, Vienna, Austria; 5 Department of Gynecology and Obstetrics, Medical University of Vienna, Vienna, Austria; 6 Department of Internal Medicine I, Medical University of Vienna, Vienna, Austria; 7 Department of Internal Medicine III with Haematology, Medical Oncology, Haemostaseology, Infectiology and Rheumatology, Oncologic Center, Paracelsus Medical University Salzburg, Salzburg, Austria; 8 Salzburg Cancer Research Institute-CCCIT, Salzburg, Austria; 9 Cancer Cluster Salzburg, Salzburg, Austria; 10 Department of Surgery, Ordensklinikum Linz - Sisters of Charity, Linz, Austria; 11 Department of Gynaecology, Medical University Innsbruck, Innsbruck, Austria; 12 Center for Cancer Research, Medical University of Vienna, Vienna, Austria; 13 Department of Surgery, Regional Hospital Wiener Neustadt, Wiener Neustadt, Austria

**Correction to: Breast Cancer Research and Treatment** 10.1007/s10549-023-07186-6

In this article, the order that the author Richard Greil appeared in the author list was incorrect and below two minor errors were missed out by mistake.

1. In Methods under Abstract “0a6A” should have read “06a”
2. In the last line of Introduction “-06A” trial should have read “06a”

The original article has been corrected.

